# University management nurse: a grounded theory

**DOI:** 10.1590/1518-8345.2199.2980

**Published:** 2018-03-08

**Authors:** Kamylla Santos da Cunha, Selma Regina de Andrade, Alacoque Lorenzini Erdmann

**Affiliations:** 1 Doctoral student, Universidade Federal de Santa Catarina, Florianópolis, SC, Brazil. Scholarship holder at Conselho Nacional de Desenvolvimento Científico e Tecnológico (CNPq), Brazil.; 2 PhD, Adjunct Professor, Departamento de Enfermagem, Universidade Federal de Santa Catarina, Florianópolis, SC, Brazil.; 3 PhD, Full Professor, Departamento de Enfermagem, Universidade Federal de Santa Catarina, Florianópolis, SC, Brazil.

**Keywords:** Grounded Theory, Faculty, Nursing, Schools, Nursing, Education, Nursing, Organization and Administration, Universities

## Abstract

**Objective:**

to understand the meaning of the university management performed by nurses managers of the nursing undergraduate course of a public university.

**Method:**

this is a qualitative research, based on the grounded theory. Data collection took place between May and September 2016, with open interviews, in the scenario of a federal public university. The technique of constant comparative analysis of the data was followed, obtaining a theoretical sample with 19 nurses, in two sample groups.

**Results:**

there were three categories emerged that shaped the phenomenon: Articulating complex collectives through university management for the qualified training of new nurses. The categories included: a) conditions, defined by perceiving the commitment to the collective, previous experiences, and training for health management, as motivations to be a teacher manager; b) actions/interactions, delimited by Knowing and recognizing, in practice, the university management process, limits and possibilities in the coordination of complex collective subjects; and, c) consequences, such as Improving teaching work and taking responsibility for university education.

**Conclusion:**

the nurses teaching managers to explain university management as a set of individual and collective actions that, articulated in a complex social environment, promote conditions for the training of critical and reflexive nurses with the demands of society.

## Introduction

The university management has a set of administrative actions that permeate specific activities related to the functioning of the higher education institution. Universities differ from other educational institutions, mainly because of the indissociability of teaching, research and extension, and constitute structures and processes that influence and are influenced by political, economic and cultural contexts^1^.

In the organizational process of university management, there is a provision for management positions held by university professors and, in the case of nursing training, by teaching nurses, who often did not have a prior preparation for this purpose^2^. When focusing on postgraduate education in nursing, based on teaching and research, the focus tends to management in health and nursing, not to the management of university structures, nor to the practices that permeate these structures, which form the teaching nurses^3^.

The lack of preparation in contents of university management can trigger overload of the teaching work and difficulty of management when performing management activities, to the detriment of those of teaching, research, and extension. However, when understanding that experience in these positions adds positive values to personal and academic life, the teacher recognizes and expands his knowledge for the functioning of all spheres of the university^4^ ^-^ ^5^.

International studies reveal the relationship between satisfaction and permanence in the work of teaching nurses^6^ ^-^ ^9^. Due to the multiple responsibilities that these professionals assume in developing university activities and to the conflicts of roles, stress, and demotivation in remaining in higher education, it is evident that there is a greater dissatisfaction of the nurse teacher to act in administrative structures and administrative processes. In Brazil, a recent study showed that the discussion about the multiplicity of activities carried out by nursing professors, including administrative positions in universities^10^, is still at an early stage.

It should be mentioned that national and international realities are different in the form of organization and specialization of teaching work. In some European countries^11^, the hiring of teachers can be carried out according to professional experience and qualification, is possible to develop specific teaching, research or university management activities according to their interest, without impeding the development of the other. Differently, in Brazilian universities, based on the evaluation, regulation, and supervision of performance policies, the teaching performance carries out teaching, research and extension activities and at some stage of his career, he will assign university management activities^12^ ^-^ ^13^.

Considering that teaching practices need to recognize management activities developed in a university environment; that such practices require efficiency to overcome challenges of their attributions; and that the understanding of the context of university management exercised by nurse teacher is still incipient, the question is: What is the meaning of university management, carried out by teaching nurses managers of an undergraduate nursing course of a public university? I aim to understand the meaning of the university management carried out by teaching nurses managers of the undergraduate nursing course of a public university.

## Method

This is a qualitative research with the methodological support of Grounded Theory (GT), updated Straussian strand^14^, seeking to understand social phenomena from the meanings of relationships and interactions between people.

The scenario studied was the Nursing Department of a public university in the south of Brazil, and the data collection was carried out from May to September 2016, through open and individual interviews, recorded in voice digital audio recording with duration average of 40 minutes. Participants were invited via email, with all interviews previously scheduled and held in the workplace.

The theoretical sample consisted of 19 nurses, university managers distributed in two sample groups^14^. The inclusion criteria for the sample groups were: university teaching nurses; in the Department of Nursing with exclusive work regime; active or retired, occupying university management positions (bosses or ex-bosses, coordinators or former course coordinators, and teaching nurses in other university management positions). The exclusion criteria for both groups were: ex-teachers managers away from work for any reason during the data collection period and substitute teachers. The first sample group was chosen intentionally due to the effective exercise of the departmental command. Based on their responses to a broad and central question, new questions emerged, directing data collection to the second group, based on the formulation of a hypothesis.

The data collection, analysis, and categorization stages were simultaneously through the constant comparative analysis of the data^14^. During the analysis of the first sample group, it was identified that the university management process by the teachers was based on three distinct but complementary realities: departmental management (related to the nursing department), teaching management (encompassing nursing course activities) and institutional management (linked to the organizational structures of the university as a whole). The data allowed denominating the first ones - departmental management and teaching management, like micromanagement; and, institutional management as a micromanagement.

**Figure 1:**
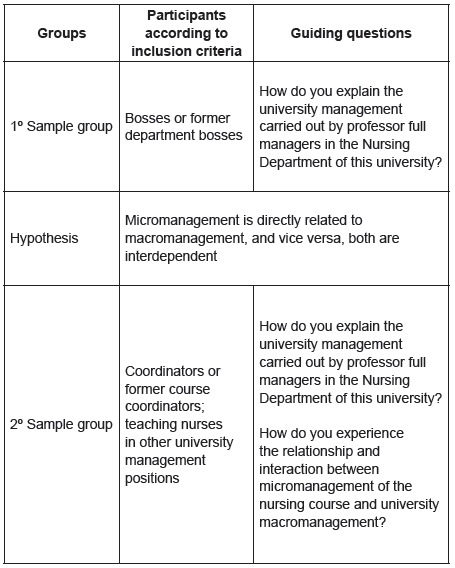
Composition of sample groups, hypothesis, and guiding questions

The analysis of the data of the first group had the following hypothesis: Micromanagement is directly related to macromanagement, and vice-versa, both being interdependent, leading to the new data collection, originating the second sample group. In this, teaching nursing managers acting both in micro and macro management were interviewed. Figure 1 illustrates the composition of the sample groups, hypothesis and guiding questions.

The analysis process followed the open, axial and integration coding. In the open coding, the data were analyzed line by line to identify each incident. Codes were generated that, after grouping, promoted the elaboration of the concepts. In the axial codification, the data were regrouped, aiming to obtain a clearer and more complete explanation about the phenomenon, associating categories to its subcategories by a systematized analytical process of comparison and connection, guided by the paradigmatic model of three components of the updated version of this strand^14^: (1) “condition” - answers questions about why, when and how a given phenomenon happens, designated by the action; (2) “action/interaction” - it is the express response of events or situations that in some way contributes to give meaning to the movements (strategies and intervening factors) that define the object of study; (3) “consequence” - which expresses expected or actual outcomes and results, effects of actions and interactions^14^.

Finally, in the integration phase, the categories and sub-categories found were compared, analyzed and refined^14^, emerging the phenomenon titled: “Articulating complex collectives through university management for the qualified training of new nurses.” Saturation theoretical data was achieved with the repetition of information about the phenomenon and absence of new elements relevant to the study objective. Memos and diagrams^14^ were elaborated on the insights of the researchers in the process of constructing the theory. NVIVO® software was used to organize the data during the analysis and coding phase.

This study complied with the ethical precepts of Resolution n. 466/2012 of the National Health Council. The project was approved by the Committee of Ethics in Research with Human Beings of the Federal University of Santa Catarina, under Opinion number 1,468,660 and Certificate of Presentation for Ethical Assessment n º (54254116.1.0000.0121). To ensure the confidentiality and anonymity of the participants, the letter E followed by the number corresponding to the order of the interviews to designate them (E1, E2 ... E19) and the indication of the sample group - first group (G1) and second group (G2) - as follows: (E1G1); (E1G2).

## Results

The theoretical sample of this study was composed of 19 teaching nurse managers. Seven former department bosses and two acting department bosses participated in the first sample group; and, four former course coordinators, four ongoing course coordinators and two teaching nurses who were in management positions in other university bodies composed the second sample group.

**Figure 2:**
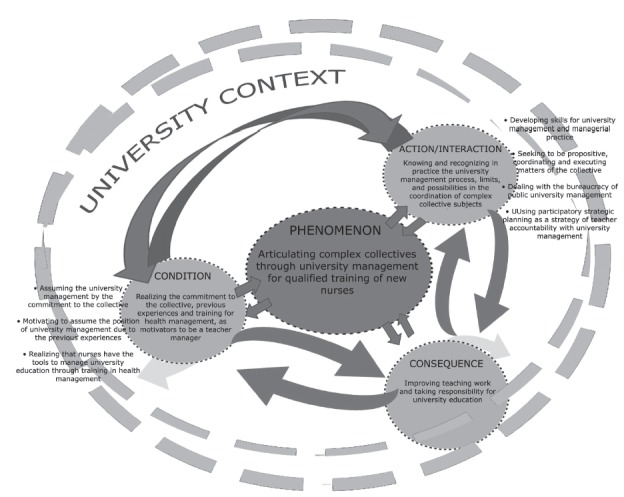
Phenomenon: Articulating complex collectives through university management for qualified training of new nurses

The phenomenon of the study supported by three interrelated categories emerged from the process of analysis and systematic integration of data, as shown in Figure 2.

The component *condition* - “Realizing the commitment to the collective, previous experiences and training for health management, as motivators to be a teacher manager” is supported by three subcategories, and promotes a movement inducer to the central phenomenon.

In the first subcategory - “Assuming the university management by the commitment to the collective”, the participants emphasized that in the university studied, the university management positions of the Nursing Department are occupied by professor nurses. At some point in their university career, they feel motivated to take up management positions, for professional commitment to other teachers, students, and university.

*I took the management position as a very big commitment. An ethical, moral, professional, personal and collective commitment, since we are dealing with the destiny of a teaching department [...] a teaching department offers the knowledge to train people who will become professionals and who will occupy spaces in society, being able to be an assistant nurse, chief, politician. (E7G1)*

The subcategory - “Motivating to assume the position of university management due to the previous experiences”, evidences that such experiences, in public or private educational institutions, hospitals and/or public secretaries enabled to know the managerial tools, stimulating them to assume the management positions in the context of the public university.

*The previous experiences contributed to my ability to carry out the management, as I did not have any formal training here, but I had previous management experiences and this instrumented me and left me with the conditions to understand that the management tools are the same as any and can be applied at the university. (E13G2)*

- “Realizing that nurses have the tools to manage university education through training in health management” is the subcategory in which the participants showed the interface with health management of their training. With this, they were instrumented with fundamentals and management principles, which made them able to manage people, conflicts, resources, materials, and infrastructure.

*What happens is that the nurse has the management in their training, the management of care, health services and with a strengthened base ... of course that both the university has its specificity, as the public health service has, as the private service has its specificity, but this guides the management exercised by him in the university. (E9G1)*

The action/interaction component - “Knowing and recognizing in practice the university management process, limits, and possibilities in the coordination of complex collective subjects”, is supported by four subcategories that represent the movements, relations, and interactions realized by the participants, given the meanings are given to the conditions related to the phenomenon.

In the first subcategory - “Developing skills for university management and managerial practice”, the participants pointed out that knowledge of the structure of the nursing department and university is important, and positively influences their abilities for university management. Knowledge of managerial processes has developed through their experiences, especially when traversing university interactive paths and acquiring significant mastery of university legislation.

*I believe that I have learned a lot from practice, from day to day to understand the whole administrative process [...] you end up realizing the importance of university management for our day to day teaching, because depending on the attitude you take it repercussions throughout an entire service network. (E11G2)*

The subcategory - “Seeking to be propositive, coordinating and executing matters of the collective” reveals that to ensure the quality and excellence of the work in university management, it is necessary that the manager understands the different roles of the people in the institution. Also, the perception of their own performance in this process allows for indispensable conditions of approach, planning, and intervention, to outline strategies for the development of collective action. The work of the manager becomes complex since it develops structural, political, economic, human, pedagogical and awareness issues of all the parties involved in the university context. It is a question of reconciling multiple interests, which are not always convergent, bringing together collective ideas.

*In university management, you are currently coordinating a group so that the group puts their ideas together ... ideas must always be in the group because no one is in charge of anybody and you [teacher manager] are coordinating a group and doing that things happen to everybody like it. (E6G1)*

In the third subcategory - “Dealing with the bureaucracy of public university management”, participants pointed out that before they took up the position of university manager they had little idea of the challenges. By exercising this position, they recognized that the bureaucratic limits are much more complex than they imagined. An example of this is in the means to obtain administrative-technical staff, teaching materials and infrastructure, which besides to difficult funding, it follow many rules and legal steps, understood only when a managerial position is occupied. Issues that could be resolved quickly turn out to be lengthy, considering the bureaucratic and legal conditions of a public university.

*At the public university, everything is done by bidding. If the air conditioning is damaged, I cannot call a private company to come fix it, I have to call the company that won the independent bidding if it is not the best company [...] everything that implies in bidding, if a bidding is not made well done, well tied up, you give way to competing companies that are not so qualified to compete for that service and then they earn because the value is lower, because, in the university, the bidding wins for those who have the lowest price and not necessarily, the one that has the best quality. (E13G2)*

The autonomy for decision making of the teaching managers was also relativized by the participants since the university context has many legal, hierarchical and financial limits. They reported on the need to increase a proactive movement of the teaching manager so the work is not plastered by the dependence of university macromanagement. To this end, they recommend dialogue and the establishment of constant partnerships, respecting the hierarchical structure of the governability instances of the institution.

*In this space of autonomy we can do a lot of things, we can do a job only to respond to what the demands are, be a manager who administers bureaucratically the demands that come to you, as a manager, or a manager can be proactive, think in the complexity of work, to have a look at all those who carry out that work, and also, have to have a look at who is the object of our work. (E3G1)*

An important strategy to solve the lack of material resources and infrastructure available by the university was to make teachers responsible for attracting financial resources. This action demands a programming to contribute to the development of the collective work and qualification of the formation of graduates to the teachers. Accountability is not mandatory, but understood as necessary, given the situation of financial constraint that public universities are experiencing today. The teaching manager uses strategies to raise awareness among other teachers in search of edicts and in the preparation of good projects to compete for funding. No less relevant, conscious responsibility for scientific research and production and ongoing curriculum upgrading are also encouraged, since the evaluation of projects by development agencies includes this global perspective of qualification.

*We have this structure because some teachers or groups of teachers have made an effort in their research project, because not everything that is here, it was not bought by the university. Teachers do projects to develop their research and to benefit the research groups they work for, so deep down, the benefit is always for the collective. (E12G2)*

The fourth subcategory, “Using participatory strategic planning as a strategy of teacher accountability with university management,” evidences the use of this method of planning by the managing teachers, as a strategy to involve other teachers in the administrative life, in order to become with university management to achieve short, medium and long-term goals.

*At my conception, she should work with peers because the more she can engage the peers, the more these people will appropriate the knowledge and then you will have a much greater strength. If the boss does everything alone, without the participation of the peers, you will not have dialogue, force ... Now, if you have a support group, this support group, even, can be together in these instances. For example, a discussion with the coordinator of the teaching department that distributes the teachers, if I have a group of coordinators that is well-tuned, I can also call this person to talk and we know that the coordinators will help and reinforce what the boss is saying. (E2G1)*

Difficulties to understand the dimensions of the university management process by the teachers were identified by the participants of this study. In general, lack of understanding denotes a reduced view of the university, restricted only to classroom space, unrelated to all the processes necessary for its microspace to function.

*Graduation students rarely perceive the university as a whole, they only perceive this space in the classroom. He does not have this notion or the higher idea of the university, not just the undergraduate, but many teachers also have this difficulty of understanding the size of the university’s work beyond this classroom space. (E12G2)*

The component *consequence* - “Improving teaching work and taking responsibility for university education” constitutes a unique category and represents the real outcomes and repercussions of the actions and interactions related to the phenomenon.

The participants emphasized that their experience in university management contributed to the improvement of their teaching work since it enables to broaden the view of the university and of the collective context. This experience was evaluated positively by them.

*This idea of the collective that you come to have - because sometimes you have only vision of your discipline, your classroom space - and you can perceive, as it articulates the different elements that pass in this space teachers, students, staff and how the task or role of each one in this process is extremely interdependent. (E14G2)*

*If I looked back I would do it all over again. I think my management experiences were super important. They determined my own academic trajectory. (E6G1)*

At the same time as the experiences of teaching nurses as managers, they allowed broadening their labor perspective to the structures that make up the institution. They also demanded to incorporate new competences to develop their activities with the collective in a competent way, adhering to social and educational policies.

## Discussion

The findings of this study have intentions that led the teaching nurses to assume positions of university management and their relationships and interactions with the teaching structures. Furthermore, they recognized bureaucratic aspects of the management process and highlighted strategies to assess the viability of their work, their peers and all those involved in the qualification of new nurses.

The interface with the health management that the undergraduate nursing course has is reported by the teaching nurses as an instrumental strength, based on the fundamentals and principles of management. The training of nurses focused on the development of managerial competences are elements such as decision making, leadership, communication, workforce management, management of physical resources, materials and information^15^. The development of these elements enables professionals to develop inventiveness and reflection when carrying out managerial practices in different contexts, as well as those of university teaching.

The aim of this study was to analyze how the ideal and real managerial competences of Nursing undergraduate coordinators identified positive issues related to leadership capacity, participatory management and good interpersonal relationships that contributed to the formation of students^16^. A study conducted in the United States of America with nursing professors showed that the leader, committed to the collective, is decisive in providing means for the development of academic activities and encouraging greater professional satisfaction among those involved being a fundamental support^17^.

When seeking to describe the development of skills for managerial practice, the participants admitted that there are specificities of university management recognized and developed in daily practice. In this way, the professors assume the management positions without having specific knowledge or initial abilities for the exercise of the function, learning and being instrumentalized throughout their professional activity, observing daily labor processes and making empirical attempts that can be positive or negative depending on their errors and correctness^18^.

In fact, the participants declare the importance of learning in the daily practice of management in trying to overcome the demands imposed by the university context, but sustain the need for specific instrumentalization, through permanent education, so they can move forward and go beyond improvisation and creativity. A case study, with the purpose of reflecting on the importance of previous training to teachers who undertake administrative activities, describes bureaucratic referrals as one of the greatest challenges currently found in public university management^10^.

The reports evidenced participatory strategic planning as a strategy launched by the participants to deal with the bureaucracy found in the management processes. A study carried out at a university in the south of Brazil elucidates that collective, articulated and participatory work fosters new relationships and interactions to make safer decisions about the technical, administrative, didactic and pedagogical aspects aimed at attending the curricular dynamics, the demands teachers, students and the institution^19^.

Also referring to the challenges found by teachers when they hold university management positions, international studies launch mentoring programs that can serve as inspirations in preparing teachers to overcome the slowness of university administrative paths. These programs can be an efficient strategy of support between a mentor teacher and an apprentice teacher by encouraging, motivating, providing support and assisting professional growth, guiding the best administrative paths to be followed^20^ ^-^ ^21^.

Regarding the repercussions of university management, the participants of this study emphasized that the management experience enabled the improvement of their teaching work. The teacher’s experiences in university management had positive repercussions on his position as an educator and helped in his understanding of university structures^5^.

The quality university education is, in fact, the product of all the efforts made by the teaching nurse managers. They act indirectly in the care of patients and the community, but they play a fundamental role in developing an expanded, shared and articulated view to promoting the quality of nursing courses^22^. Thus, when recognizing the efforts of the teaching staff nurses, it is evident that university management is feasible when linked to the establishment of horizontal relationships and actions based on dialogue, respect, and understanding of the role of each involved in ensuring the training of nurses qualified staff.

This study is limited by understanding the meanings of university management performed by teaching nurse managers in only one university setting. The scarce national literature of studies with similar focus was also a restriction found at the time of the discussion. Considering the complexity of this issue, the results of this study contribute to the reflection and discussions of teaching nurses and managers from other public and private university contexts emerging needs of national and international requirements for the management of higher education and the quality of nursing courses.

## Conclusion

The findings of this study evidenced that the university management carried out by teaching nurse managers refers to a set of individual and collective actions. These actions are diluted in a social space in which specific, singular and complex groups are articulated, in constant relationship and interactions, seeking to overcome the bureaucratic managerial assumptions and the slowness of university management processes. The product of all the efforts expended by nurses teaching managers is a quality university education for the excellence of future nurses.
